# Giant Left Atrial Pleomorphic Sarcoma With Echocardiographic Characteristics Mimicking a Myxoma

**DOI:** 10.14740/jmc5212

**Published:** 2026-01-13

**Authors:** Madeline Castella-Chin, Kayla Canteras, Christopher Cullom

**Affiliations:** a Department of Anesthesiology, Loma Linda University Medical Center, Loma Linda, CA 92354, USA

**Keywords:** Primary cardiac mass, Atrial mass, Sarcoma, Anesthetic induction

## Abstract

Primary cardiac tumors are rare. Cardiac tumors of substantial size may present in the perioperative setting urgently, bypassing conventional imaging, thus relying on echocardiography for characterization and operative guidance. We report a unique case of a 66-year-old female with a large left atrium mass, who presented with worsening dyspnea and fatigue, with biopsy suggesting a primary cardiac sarcoma. This case is unique, as approximately 70% of left atrial masses reported in the literature are cardiac myxomas, whereas the most common site in which cardiac sarcomas develop is the right atrium. In this particular case, the location of the left-sided sarcoma resulted in mitral valve and left ventricular outflow obstruction, as well as severe pulmonary hypertension, leading to complicated anesthetic induction during surgery. This rare case of a primary cardiac tumor highlights the sequelae of obstructive atrial masses, which potentially resulted in cardiovascular collapse with induction of anesthesia. It is especially unique for pathologic findings suggesting a cardiac sarcoma. This case provides an opportunity to discuss diagnostic challenges for patients with complex pathophysiology and contributes to the limited collection of literature on cardiac sarcomas located in the left atrium.

## Introduction

Primary cardiac tumors are a rare finding and account for 0.001% to 0.03% of all cardiac tumors [1]. About 25% of primary cardiac tumors are malignant, and 75% of malignant primary cardiac tumors are sarcomas [2]. Regarding primary malignant cardiac tumors, angiosarcomas (43.2%) are the most common histologic type, followed by leiomyosarcoma (6.6%), and undifferentiated pleomorphic sarcoma (5.9%) [3]. Most malignant primary cardiac sarcomas are found in the right atrium and are predominately angiosarcomas [2]. In contrast, malignant tumors located in the left atrium are usually pleomorphic sarcomas and leiomyosarcomas [2]. The shape and location of these malignant left atrial masses contributed to misdiagnosis as myxomas [4]. Clinical presentation of left-sided masses ranges from heart failure, dyspnea, acute pulmonary edema, syncope, embolization, constitutional symptoms, and fatigue [4]. Due to nonspecific presentation of cardiac masses and the rapid growth of tumors, diagnosis can be delayed until patients are extremely unstable. Specifically in sarcomas, 66-89% of patients present when the mass has already become metastatic [5, 6]. Surgical resection of malignant tumors is recommended but is often technically difficult due to local invasion of cardiac structures. Incomplete resection is associated with substantial mortality; 90% of patients survive less than 1 year. This stands in contrast to benign cardiac tumors, which have a favorable prognosis even with incomplete resection [5]. Inducing anesthesia in patients with atrial masses requires consideration of the pathologic manifestations of atrial obstruction, and potentially, atrioventricular valvular obstruction [7]. The authors present a case highlighting the diagnostic challenges associated with cardiac masses in which medical urgency replaces traditional diagnostic modalities. Written authorization for publication of this case report was obtained from the patient in accordance with the Health Insurance Portability and Accountability Act.

## Case Report

**Figure 1:**
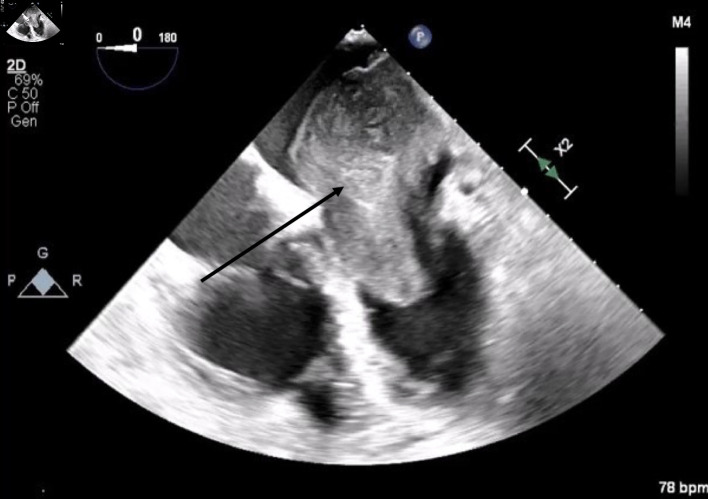
Mid-esophageal four-chamber view. A mass with lateral wall stalk prolapses through mitral valve during diastole. The arrow denotes the large cardiac tumor.

**Figure 2:**
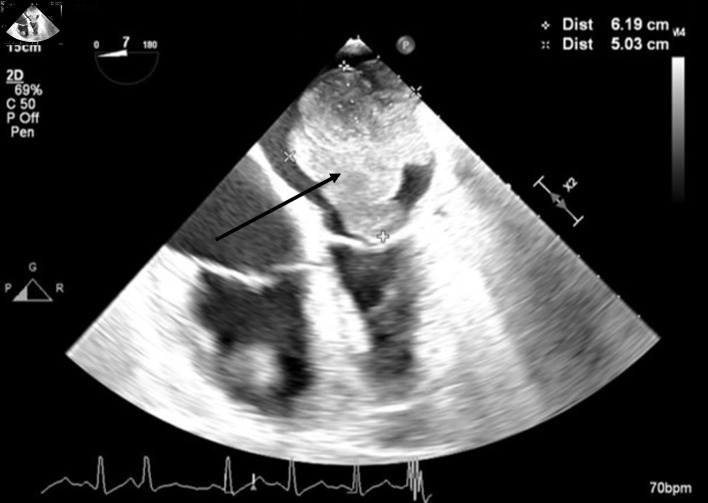
Mid-esophageal four-chamber view. A mass occupies the majority of the left atrium during systole. The arrow denotes the large cardiac tumor.

**Figure 3:**
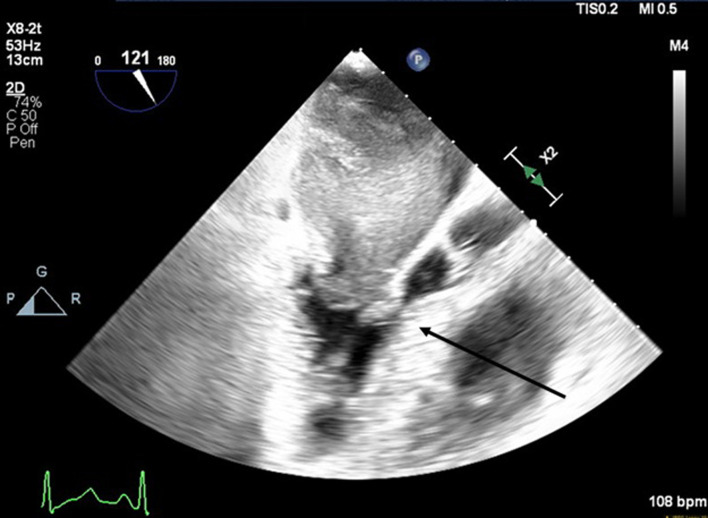
Mid-esophageal aortic long-axis view showing a mass obstructing the left ventricular outflow tract (LVOT) during early systole. The arrow indicates LVOT obstruction.

**Figure 4:**
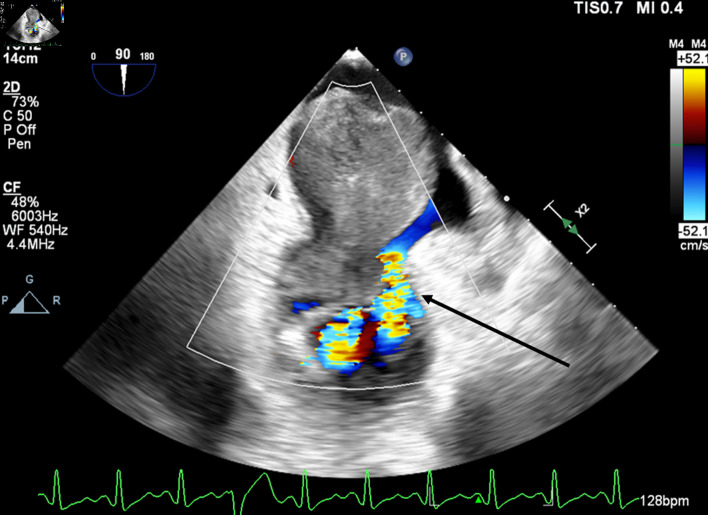
Mid-esophageal two-chamber view with color flow Doppler demonstrating turbulent flow around the mass through the mitral valve during diastole. The arrow indicates turbulent flow through the mitral valve.

**Figure 5:**
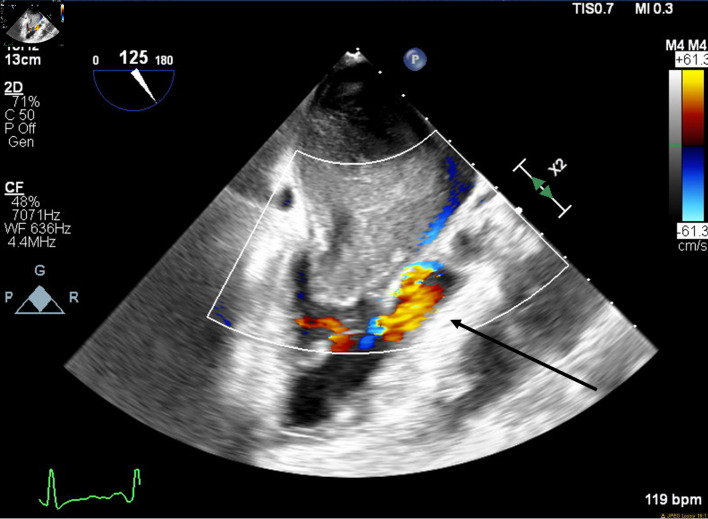
Mid-esophageal aortic long-axis view with color flow Doppler demonstrating turbulent flow through left ventricular outflow tract (LVOT) during early systole. The arrow indicates turbulent flow through the LVOT.

A 66-year-old obese woman presented to the emergency department (ED) with a 2-month history of progressive fatigue, 20-lb weight loss, orthopnea, wheezing, chest pressure, lower extremity edema, dyspnea on exertion, and paroxysmal nocturnal dyspnea. On examination, she had a systolic murmur. Transthoracic echocardiography (TTE) revealed a 5-cm mass occupying her left atrium. Right and left heart catheterization showed no flow-limiting coronary artery disease. Right heart pressures were as follows: right atrium 3 mm Hg, right ventricle 66/4 mm Hg, pulmonary artery 67/25 mm Hg, pulmonary capillary wedge pressure 25 mm Hg, and left ventricle 103/9 mm Hg. With plan for further workup, she was discharged and scheduled for elective resection. She returned to the ED 5 days later with severe acute hypoxic respiratory failure. On admission, vitals were as follows: heart rate (HR) 129, respiratory rate (RR) 44, SpO_2_ 92%, and blood pressure (BP) 110/70. Her hypoxemia was managed with high-flow nasal cannula, and she was scheduled for urgent resection of her left atrial mass. In the operating room, she was severely orthopneic when the head of her bed was lowered below 90°. A radial arterial and internal jugular central venous catheter were placed while she was in a seated position on high-flow nasal cannula (fraction of inspired oxygen (FiO_2_) 100%, 35 liters per minute (LPM)). Femoral central venous and arterial cannulas were also placed prior to induction of anesthesia to enable emergent transition cardiopulmonary bypass (CPB). A dexmedetomidine infusion was used to facilitate vascular cannulation, surgical preparation, and draping. Vasopressin, epinephrine, and norepinephrine infusions were started preemptively, and she received a 250-mL albumin bolus. Her airway was secured with video laryngoscopy after induction with etomidate and rocuronium. After induction, she became profoundly hypotensive and hypoxemic, with mean arterial pressure (MAP) dropping by 30 mm Hg and oxygen saturation dropping to 70%. This brief episode resolved with administration of crystalloid, vasopressin boluses, and Trendelenburg positioning. Despite a transient period of hypotension and hypoxia, emergency CPB was not required. A median sternotomy was performed, and she was transitioned to CPB by aorto-bicaval cannulation. Intraoperative transesophageal echocardiography (TEE, X8-2t, EPIQ CVx; Philips Ultrasound, Bothell, WA) redemonstrated a large pedunculated mass traversing the interatrial septum and occupying the right and left atria. Mid-esophageal views were used to image the large heterogeneous mass (Figs. 1, 2). She was found to have a 7 × 4.5 cm left atrial mass arising from the lateral wall of the atrium near the left inferior pulmonary vein. Pulmonary venous return was obstructed, likely contributing to her pulmonary hypertension, pulmonary edema, and hypoxemia. During atrial systole, the mass traversed the mitral valve orifice and obstructed mitral inflow and the left ventricular outflow tract (LVOT). LVOT obstruction persisted during early ventricular systole, and flow acceleration was observed with color flow doppler (Figs. 3-5).

**Figure 6:**
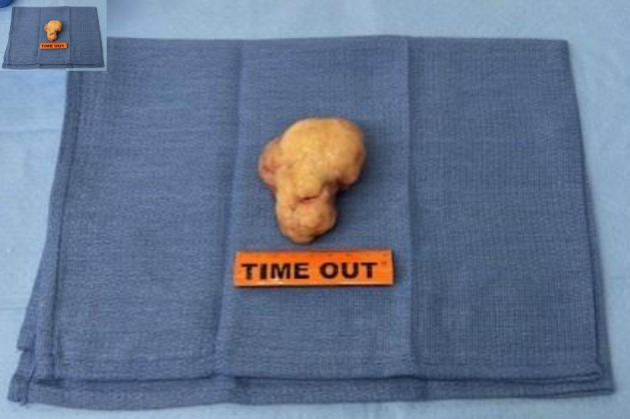
Photograph of the mass following resection.

**Figure 7:**
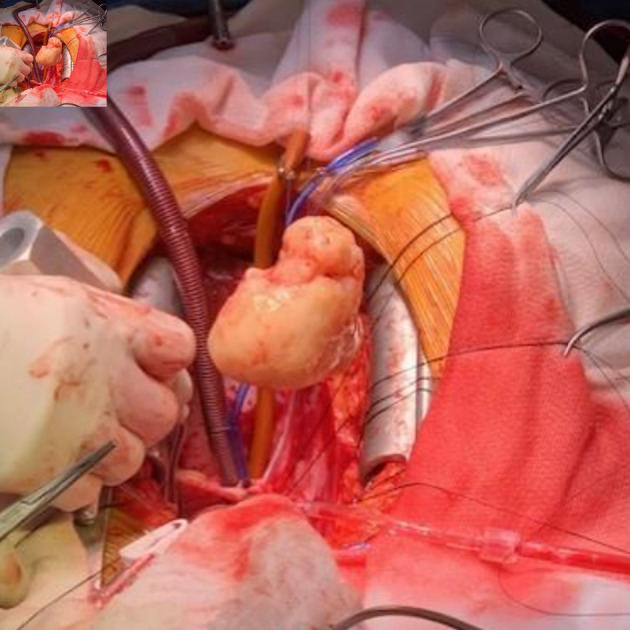
Photograph of the mass following resection during surgery.

**Figure 8:**
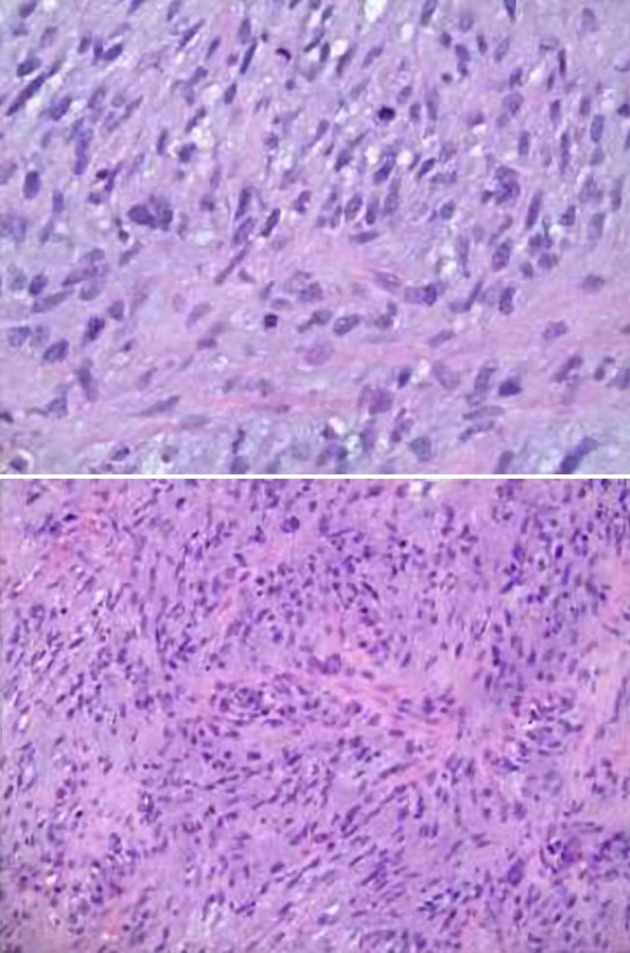
Microscopic pathology images demonstrating cardiac undifferentiated pleomorphic sarcoma (UPS), grade 3. The histology displays variable cellularity, with areas of marked pleomorphism, as well as foci of myxoid change and focal necrosis. Mitoses are easily identified in some areas and are scarce in others. These features support the interpretation of an UPS.

The mass was accessed via a right atriotomy, the fossa ovalis and intra-atrial septum were divided. The sessile base of the mass was densely adherent to the posterior wall of the left atrium proximal to the left inferior pulmonary vein. The mass was excised without fragmentation; however, a large portion of the posterior left atrium and interatrial septum was also resected and repaired with bovine pericardium. Separation from CPB was unremarkable. TEE demonstrated a normally functioning mitral valve and preserved left ventricular (LV) function. Pathologic examination showed an undifferentiated pleomorphic sarcoma with an aggregate mass of 77.8 g and maximum dimensions of 6.7 × 6.1 × 4.4 cm (Figs. 6-8). Her postoperative recovery was unremarkable, and she was discharged on postoperative day 6. Unfortunately, she suffered from tumor recurrence and died almost 2 years after resection.

## Discussion

In the setting of cardiac tumors, TEE is highly useful to better characterize the mass in terms of size, morphology, attachment site, extension, and hemodynamic effect [8]. Myxomas are the most common primary cardiac tumor; however, other masses can frequently be found within the heart, such as lipomas, sarcomas, fibroma, and rhabdomyoma. Thus, being able to differentiate between masses will guide management intraoperatively.

Echocardiographically, myxomas demonstrate a globular, pedunculated nature with an insertion point at the interatrial septum [9]; whereas the broad-based attachment and heterogenous echogenicity of sarcomas may help differentiate sarcomas from other cardiac tumors. Hypoechoic areas may indicate tumor necrosis [8]. Frequently, undifferentiated sarcomas are found in the left atrium. They are white, flesh-like tumors with distinct areas of necrosis and hemorrhage [8]. Nonetheless, left atrial sarcomas are often mistaken for myxoma by echocardiography. The most common type of sarcoma is angiosarcoma, accounting for approximately 76% of all cardiac sarcomas [6]. Angiosarcomas are typically characterized as a lobulated mass with an area of heterogeneous necrosis or hemorrhage with no stalk present. This characteristic distinguishes it from myxomas, which normally originate from the atrial septum by a narrow stalk [8]. Contrast echocardiography may not often display significant enhancement on imaging due to the slit-like vascular channels and spindle cell regions [8]. Angiosarcomas frequently take over the right atrial wall, therefore occupying the cardiac chamber [8]. Angiosarcomas may also involve the right atrioventricular groove, thus affecting the function of the tricuspid valve [8]. The resulting signs and symptoms include dyspnea, fatigue, congestion, and pericardial chest pain [8].

The second most common primary cardiac sarcoma is the rhabdomyosarcoma, which may arise from any cardiac structure with no location preference [8]. Rhabdomyosarcomas result in obstruction and can grow rapidly with early invasion of the pericardium [8]. Less common sarcomas include fibrosarcomas, leiomyosarcomas, and osteosarcomas [8]. Fibrosarcomas are characterized by white, flesh-like tumors with necrotic and hemorrhagic regions [8]. Leiomyosarcomas and osteosarcomas are commonly located in the left atrium [8]. Generally, sarcomas grow rapidly and broadly with early metastatic activity [8].

Particular consideration should be given to the striking similarity between myxoma and undifferentiated pleomorphic cardiac sarcoma (UPCS). Classic imaging workup for a cardiac mass should include TTE/TEE, chest X-ray (CXR), computed tomography (CT), cardiac magnetic resonance imaging (MRI), and fluorodeoxyglucose positron emission tomography (FDG-PET) [10, 11]. PET can help with differentiation as the accuracy of malignancy detection with PET exceeds 96% [11]. Even with advanced imaging, it has been reported that undifferentiated sarcomas may continue to masquerade as myxoma. Pathologic examination may be required for diagnosis [10]. Management of undifferentiated pleomorphic sarcoma differs from that of myxoma; and pathologic distinction is important. Ultimately, a malignant diagnosis leads to a more extensive and complete resection [10].

Left atrial sarcomas also manifest with the same dynamic obstructive pathology as myxoma. Dynamic mitral valve obstruction can impede ventricular filling, and when obstruction persists during ventricular systole, cardiac output may be compromised. Similar to the management of mitral stenosis, it is critical to maintain a heart rate that allows adequate LV filling time, in addition to maintaining sufficient LV preload [7]. Moreover, left atrial masses may impede pulmonary venous drainage promoting pulmonary hypertension and pulmonary edema [7]. Respiratory failure further complicates anesthetic management. Preparation for emergent transition to CPB should be considered for patients with significant obstructive pathology or respiratory decompensation prior to induction of anesthesia.

Precipitous decompensation may preclude definitive diagnosis of malignancy prior to resection. Although rare, broad-based masses that do not arise from the fossa ovalis should increase the index of suspicion for malignancy. Gross intraoperative examination and biopsy for pathologic examination may be warranted. However, even an undifferentiated sarcoma can have a stalk and arise from the interatrial septum [1]. Other TEE findings that favor malignancy include infiltration, irregular margins, necrosis, pericardial effusion, and involvement of more than one chamber [10]. As Valles-Torres et al [1] demonstrated, ultimately no imaging modality can differentiate every UPCS from a myxoma in all situations thus highlighting the importance of intraoperative histopathology. Malignant diagnosis has the potential to alter surgical resection if practical.

Malignant primary cardiac tumors have a very poor prognosis. Without surgical resection, only 10% of the patients will survive 12 months. This case highlights the complexity of diagnosis of atrial masses and sequelae of dynamic intracardiac obstruction. Atrial masses may impair pulmonary venous return, LV filling, and rarely, LVOT outflow. Diagnosis of primary cardiac malignancy often requires a high index of suspicion. Precipitous decompensation may curtail preoperative evaluation, and workup should include CT, MRI, and TTE to fully characterize the mass, which may provide enough evidence to suggest whether the mass is potentially malignant or not. However, there are situations where medical urgency may supersede imaging workup thus demonstrating the importance of the echocardiographer’s understanding of the small nuances between the two masses. Any suggestion of malignancy on intraoperative TEE can steer the surgical decision making significantly. Furthermore, the importance of intraoperative histopathology cannot be minimized due to the striking likeness between myxoma and UPCS.

### Conclusions

This case contributes to the finite collection of literature discussing large left atrial sarcomas, demonstrating a unique presentation of a large posterior wall sarcoma in a 66-year-old patient. The size of the tumor created multifaceted pathology including mitral valve obstruction (MVO), pulmonary hypertension (PH), and LVOT obstruction. The induction plan should include consideration for preemptive volume loading or hemodynamic support, preparation for conversion to CPB prior to induction of anesthesia, and consideration of the physiological balance between each pathology. Because prognosis and surgical management differ sharply between benign and malignant cardiac masses, intraoperative TEE is paramount for differentiating among the various types. The rarity of cardiac sarcomas especially with a size and location as described by the authors, represents the challenge for diagnosis and subsequent therapeutic approach.

### Learning points

Primary cardiac tumors can have a precipitous onset and can frequently require clinicians to bypass typical imaging pathways in pursuit of rapid management and stabilization, given the risk of hemodynamic compromise from obstruction, cardiac conduction implications, respiratory failure, pulmonary hypertension and right heart failure. Echocardiographic investigation is critical in evaluating the mass and its implications in the pathophysiology of each patient’s case in order to manage each patient from an anesthetic and hemodynamic perspective. However, given the varying presentation on echocardiography, it is imperative to have histopathology studies done during surgical resection to accurately diagnose and appropriately treat each mass.

## Data Availability

Any inquiries regarding supporting data availability of this study should be directed to the corresponding author.

## Abbreviations

LVOT: left ventricular outflow tract
CPB: cardiopulmonary bypass
TEE: transesophageal echocardiography
UPCS: undifferentiated pleomorphic cardiac sarcoma
CXR: chest X-ray
CT: computed tomography
MRI: magnetic resonance imaging
PET: positron emission tomography
PH: pulmonary hypertension
MVO: mitral valve obstruction
